# Comparison of error-based and errorless learning for people with severe traumatic brain injury: study protocol for a randomized control trial

**DOI:** 10.1186/1745-6215-14-369

**Published:** 2013-11-05

**Authors:** Tamara Ownsworth, Jennifer Fleming, Robyn Tate, David HK Shum, Janelle Griffin, Julia Schmidt, Amanda Lane-Brown, Melissa Kendall, Mathilde Chevignard

**Affiliations:** 1 School of Applied Psychology and Behavioural Basis of Health Program, Griffith Health Institute, Griffith University, Mt Gravatt 4122, Australia; 2 School of Health and Rehabilitation Sciences, University of Queensland, St Lucia, Australia; 3 Princess Alexandra Hospital, Wooloongabba, Australia; 4 Rehabilitation Studies Unit, University of Sydney, Sydney, Australia; 5 Brain Injury Unit, Royal Rehabilitation Centre, Sydney, Australia; 6 Australian Catholic University Sydney, Brisbane, Australia; 7 Brain Injury Rehabilitation Unit, Liverpool Hospital, Sydney, Australia; 8 Acquired Brain Injury Outreach Service, Brisbane, Australia; 9 Rehabilitation Department for Children with Acquired Brain Injury (INR-A), Hôpitaux de Saint Maurice, Saint Maurice, France; 10 ER6, Université Pierre at Marie Curie, Paris 6, France

**Keywords:** Brain injury, Metacognition, Self-awareness, Rehabilitation, Functional activities, Randomized controlled trial

## Abstract

**Background:**

Poor skills generalization poses a major barrier to successful outcomes of rehabilitation after traumatic brain injury (TBI). Error-based learning (EBL) is a relatively new intervention approach that aims to promote skills generalization by teaching people internal self-regulation skills, or how to anticipate, monitor and correct their own errors. This paper describes the protocol of a study that aims to compare the efficacy of EBL and errorless learning (ELL) for improving error self-regulation, behavioral competency, awareness of deficits and long-term outcomes after TBI.

**Methods/Design:**

This randomized, controlled trial (RCT) has two arms (EBL and ELL); each arm entails 8 × 2 h training sessions conducted within the participants’ homes. The first four sessions involve a meal preparation activity, and the final four sessions incorporate a multitasking errand activity. Based on a sample size estimate, 135 participants with severe TBI will be randomized into either the EBL or ELL condition. The primary outcome measure assesses error self-regulation skills on a task related to but distinct from training. Secondary outcomes include measures of self-monitoring and self-regulation, behavioral competency, awareness of deficits, role participation and supportive care needs. Assessments will be conducted at pre-intervention, post-intervention, and at 6-months post-intervention.

**Discussion:**

This study seeks to determine the efficacy and long-term impact of EBL for training internal self-regulation strategies following severe TBI. In doing so, the study will advance theoretical understanding of the role of errors in task learning and skills generalization. EBL has the potential to reduce the length and costs of rehabilitation and lifestyle support because the techniques could enhance generalization success and lifelong application of strategies after TBI.

**Trial registration:**

ACTRN12613000585729.

## Background

With an estimated 10 million new cases each year, traumatic brain injury (TBI) is a highly prevalent condition that results in devastating long-term consequences [1]. Over 70 % of people with severe TBI have been estimated to require long-term care and support due to relationship breakdown and the loss of independent living skills and work capacity [2]. Not surprisingly, the socioeconomic burden of TBI is enormous. In Australia, the estimated cost of severe TBI was AU$8.6 billion in 2008 alone, and average lifetime cost per person was AU$4.8 million [3].

Although individuals with TBI have diverse long-term support needs, the most challenging consequences that necessitate long-term support from families and the healthcare system are people’s impaired self-awareness, poor self-monitoring and impulsive behaviors [4,5]. Severe TBI typically damages the prefrontal brain regions and connecting pathways that regulate error self-regulation or the metacognitive capacity to stop, check and modify one’s behavior in accordance with task requirements [6]. Consequently, many people with severe TBI display impaired awareness of their deficits and make errors of judgment that reduce their safety and autonomy (for example, unsafe actions using the stove, spending beyond their budget and so on) [4,7]. Error self-regulation impairments greatly compromise safety and independence on everyday tasks and are largely responsible for the failure to transfer skills learned in a training context to other daily situations [7]. However, there are different perspectives in the literature concerning the role or function of errors in the learning process.

A dominant view in the rehabilitation literature is that people with severe cognitive impairment learn new skills more effectively when errors are avoided during training, using the ‘bottom-up’ approach of errorless learning (ELL) [8,9], as compared to trial-and-error learning. In trial-and-error learning, people are encouraged to attempt or guess responses on a task, and each time an error is committed the therapist corrects their response without providing further feedback on performance [10]. In contrast, ELL involves the therapist preventing the person from making errors during the learning acquisition phase. ELL is a recommended method for teaching task-specific skills to people with severe memory impairment after TBI [10,11]. This may occur by modeling each step and having the person practice only correct responses over sessions to support habit formation (for example, error-free recall of the steps required to send a text message). Despite achieving superior learning outcomes to trial-and-error learning, there is evidence that ELL does not elicit generalization from the training task to a novel and untrained set of procedures [8-11]. The failure to generalize skills after ELL has been attributed to an overreliance on the therapist to anticipate and prevent errors during training [11]. In particular, during ELL individuals are not provided with the opportunity to make errors and become aware of these during performance or to reflect upon their functional significance. Similarly, individuals are not taught to anticipate and self-correct their errors because the therapist closely monitors their performance and provides prompts to prevent their occurrence. As a result, similar intensive external support is required for individuals to achieve error-free performance on a new training task [10,11].

In contrast, the ‘top-down’ approach of error-based learning (EBL) provides structured opportunities for individuals to commit errors, and become aware of and self-correct these errors through graded prompts and feedback from the therapist [12]. Similar to trial-and-error learning, individuals are encouraged to attempt responses on tasks that could lead to an error. However, unlike trial-and-error learning, EBL is metacognitive approach that uses systematic feedback and graded prompts to target the following processes: (1) learning to routinely stop, check and self-correct errors on daily tasks; (2) reflecting on the meaning of errors on tasks to promote awareness of deficits; and (3) anticipating errors in everyday situations and planning strategy use accordingly [7,12]. A key premise of the EBL approach is that an internal focus on recognizing and correcting one’s own errors facilitates the spontaneous carryover of self-regulation skills to other untrained tasks and situations in daily living. Metacognitive skills, or the capacity to reflect on, monitor and regulate one’s own actions, are integral to recognizing and compensating for the frequent problems that arise in daily situations due to the effects of TBI [7,12].

Reviews have identified 12 brain injury clinical trials that compared ELL to a control condition of trial-and-error learning [10,11], whereas only 3 trials compared EBL (systematic feedback) to control conditions (that is, wait list or practice plus therapist corrected errors) [13]. ELL and EBL were each found to be more effective than control conditions. However, the relative efficacy of these two theoretically opposing neuropsychological approaches is yet to be compared.

The opposing principles of EBL and ELL and hypothesized training outcomes are presented in Table 1. Despite different mechanisms of learning, both approaches are expected to promote functional gains on the specific training task. However, by training internal self-monitoring and regulation of errors, only EBL is predicted to promote generalization of self-regulation skills to non-trained tasks when external support from the therapist is withdrawn. Further, because EBL provides opportunities for people to make errors and support to reflect upon the functional significance of these, only EBL is expected to increase people’s awareness of their deficits. There is preliminary evidence supporting the efficacy of metacognitive approaches for improving awareness of deficits and functional task performance.

**Table 1:** Comparison of the key principles and expected training outcomes of error-based learning and errorless learning

**Key principles**	**Error-based learning**	**Errorless learning**
Training approach and model	Top-down with an internal focus (person learns to monitor and correct their own errors)	Bottom-up with an external focus (therapist monitors and eliminates the person’s errors)
Target of intervention	Increased awareness of deficits and the capacity to anticipate, self-monitor and self-regulate errors	Error-free performance on successive parts of the task through observing and practicing correct actions
Mechanisms (function of errors in learning)	A structured opportunity to make and self-identify errors with therapist’s feedback to realize the functional significance of errors, and practice of self-initiated strategies (stop, check and correct) over sessions	The therapist prevents errors during the learning acquisition phase by modeling each step and the person practices only correct responses over sessions to support habit formation
Training outcomes (pre vs post)	Reduced errors on the training task, generalization of self-regulation skills to untrained tasks (reduced errors and improved broader behavioral competency) and greater awareness of deficits	Reduced errors on the training task, minimal change in awareness, lack of generalization of self-regulation skills to untrained tasks (minimal change in errors and broader behavioral competency)

A meta-analysis [13] by our team identified that metacognitive interventions involving therapist feedback on errors had a moderate effect (Hedges’ adjusted g = 0.64) on self-awareness and a large effect on functional task performance (Hedges’ adjusted g = 0.90), relative to control conditions (that is, wait list or conventional therapy). One of these intervention studies was a randomized, controlled trial (RCT) completed by the authors [14], which evaluated different formats of metacognitive training over eight sessions. The individual occupation-based (error-based) intervention yielded greater pre/post improvements in self-awareness and behavioral competency than another active intervention (group plus individualized support) and a waiting list control condition. Furthermore, in a series of single-case experimental studies [15,16] our team found that eight sessions of EBL yielded marked improvements in error self-regulation. In a multiple baseline design, a participant with very severe TBI showed a 44% and 39% reduction in errors within two applied settings (meal preparation and volunteer work) [15]. In the second single-case experimental study [16], EBL was associated with improved error self-regulation for the two participants with severe TBI who received this intervention. Specifically, using 2 SD band analysis (see [17]), there was a significant increase in self-corrected errors and decreased reliance on therapist support during meal preparation between the baseline and treatment phases. In contrast, a third participant who received behavioral practice with errors corrected by the therapist showed a non-significant change in errors, and instead displayed greater reliance on the therapist over time to check and correct his actions during meal preparation [16]. In a recent RCT [18], we found that multimodal feedback (that is, audiovisual plus verbal feedback on task performance) was more effective for improving error self-regulation and awareness of deficits than verbal feedback or experiential feedback alone.

Overall, previous research on EBL supports that with relatively brief and structured learning opportunities people with severe TBI can improve their error self-regulation and behavioral competency and develop greater awareness of deficits. As outlined in Table 1, both EBL and ELL approaches are expected to promote functional gains on specific training tasks. However, by training internal self-monitoring and regulation of errors, only EBL is predicted to promote generalization of self-regulation skills to non-trained tasks and increase people’s awareness of their deficits. Furthermore, given the emphasis on skills generalization and broader application of strategies to compensate for cognitive and behavioral impairments in daily life, it is expected that EBL will promote greater gains in long-term functional outcomes than ELL.

### Study objectives and hypotheses

This RCT aims to address two fundamental questions: (1) is making errors actually beneficial in the learning process or is it better to avoid errors when training skills in rehabilitation? Specifically, does an EBL approach promote greater error self-regulation and self-awareness than ELL? And (2) is improvement in error self-regulation, behavioral competency and self-awareness following intervention related to better long-term social outcomes (for example, work, independence and relationships)?

It is hypothesized that, compared to participants in the ELL condition, participants in the EBL condition will demonstrate significantly greater gains in the following areas: (1) skills generalization on error self-regulation measures between pre-intervention and post-intervention assessment; (2) improvements in behavioral competency, awareness of deficits between pre-intervention and post-intervention assessment; and (3) improvements in long-term role participation and reduced support needs between pre-intervention, post-intervention and 6-month follow-up.

Secondly, it is hypothesized that greater skills generalization (that is, improved error self-regulation and behavioral competency) and pre/post changes in awareness of deficits will predict increased gains in long-term social outcomes (role participation and support needs) relative to pre-intervention assessment.

## Methods/Design

### Trial design

The study design adheres to the ‘CONsolidated Standards Of Reporting Trials’ (CONSORT) Statement and the CONSORT extension to non-pharmacological interventions [19]. The research design entails a RCT with multisite recruitment of 135 participants with severe TBI over a 28-month period (4 to 5 participants on average per month). Participants will be recruited on a consecutive discharge basis across two cities (Brisbane and Sydney, Australia) and randomly allocated using matched assignment [20]. Global neuropsychological function is potentially related to pre-intervention functioning [21], and thus the matching process is designed to ensure a relatively equal proportion of individuals in each intervention with mild to moderate and severe neuropsychological deficits. Participants’ global neuropsychological function (mild to moderate or severe deficit subgroups) will be determined prior to the randomization. The use of ELL as an active control intervention is based on both the theoretical significance of comparing outcomes of ELL and EBL and the ethical considerations of providing a ‘credible’ intervention instead of a sham or pseudotreatment [22]. This is important for both the participants and the therapists administering the intervention.

### Participants and recruitment

Potential participants will be identified by treating therapists or case managers in three large metropolitan-based brain injury rehabilitation services in Brisbane and Sydney, Australia. Patients who meet the inclusion criteria will be approached by their therapist or case manager, who will provide a brief summary of the study. If the patient is interested in taking part, he or she will be asked to provide verbal consent for the project coordinator to contact them about the study.

A standard screening process will be employed to help ensure that the intervention targets a homogenous group of participants with awareness and dysexecutive impairments across the centers. Participants from each center will be eligible for the study if they are aged 18 to 65 years, have had a severe TBI (as determined by posttraumatic amnesia duration and Glasgow Coma Scale score), are deemed medically stable and out of posttraumatic amnesia, live within a 50 km radius of each metropolitan center, and display dysexecutive impairments that warrant care and supervision. Participants will be excluded if they are deemed unable to provide informed consent, have any combination of severe behavioral/motor/perceptual/language or cognitive impairments which would preclude the ability to undertake research of this nature, and/or psychotic symptoms or severe mood symptoms not under effective management.

Based on combined service statistics and past research by the authors it is expected that approximately 60% to 70% (*n* = 348) of the broader urban-based severe TBI pool (*n* = 536) will exhibit dysexecutive impairments that require care and supervision [23,24]. A portion of these (for example, 20%) may be excluded due to prior neurological or psychiatric disorders or a combination of severe behavioral/motor/perceptual/language or cognitive impairments. As a standard screening process, treating therapists will refer people with severe TBI who exhibit marked difficulties with instrumental activities of daily living (IADLs). Suitability for the study will be confirmed at preassessment through tests of executive function and the relative and self-report on the Patient Competency Rating Scale (PCRS [25]). A five-point discrepancy has previously been used as a general cut-off to indicate impaired self-awareness in research [26]. Participants will receive a brief neuropsychological battery consisting of tests of attention (for example, Digit Span, Trails A), memory (for example, Hopkins Verbal Learning Test), and executive function (for example, Controlled Oral Word Association Test, Trails B, Hayling Sentence Completion Test). A global neuropsychological function composite will be calculated by summing and averaging age-adjusted standardized scores (see [27]), and classified as ‘mild to moderate deficit’ (<−2 SD), or ‘severe deficit’ (≥ − 2 SD).

### Ethics and procedure

The ethical aspects of this research project have been approved by the Metro South Hospital and Health Service Human Research Ethics Committee (EC00167) and the ethics committees of the University of Queensland, and Griffith University, Queensland, Australia (HREC reference number: HREC/13/QPAH/096). After the first 6 months of data collection in Brisbane, ethical clearance for the project will be extended to the hospitals in Sydney to allow multisite participant recruitment. This project will be conducted in accordance with the National Statement on Ethical Conduct in Human Research (2007) produced by the National Health and Medical Research Council of Australia.

Informed consent will be obtained from each participant prior to commencement in the study. The independent and blind assessor will visit participants in their home to conduct the pre-intervention assessment, which will involve completing a series of questionnaires and neuropsychological tests (see Measures section below). The participant’s primary caregiver will also complete a set of questionnaires. Based on random allocation, participants will receive either the EBL or ELL intervention, both of which entail an 8-week home-based training program (one 90 to 120 minute session per week) with an occupational therapist (see Intervention section). A week after the final treatment session, the blind assessor will conduct a post-intervention assessment involving readministration of specific neuropsychological tests and questionnaires. At 6 months after the post-intervention assessment, the participant will be contacted by the blind assessor for a brief telephone-based assessment of their home and community functioning.

### Randomization

Participants’ global neuropsychological function (mild to moderate deficit (<−2 SD) or severe deficit (≥ − 2 SD)) will be used to determine their subgroup for random allocation based on matched assignment. The random assignment will be conducted independent of the project staff involved in the interventions and the blind assessor. For each subgroup (that is, mild to moderate or severe deficit), participants will be randomly allocated to EBL or ELL using sequentially numbered and sealed opaque envelopes. The envelopes will contain group allocation on a written insert, based on a predetermined random computer generated sequence [20].

### Intervention procedures

Manualized treatment protocols have been developed for both interventions from pilot research. Uniform elements of both EBL and ELL include: (a) eight weekly treatment sessions of approximately 90 to 120 minutes conducted in the participant’s home; (b) use of verbal reinforcement for correct performance; (c) learning to prepare a main meal (a stir fry) for the first four training sessions (1 to 4); and (d) development of a set of errands for participants to learn in the home or community as the second multitasking training activity for the last four sessions (5 to 8). The second training activity introduced in session 5 of both interventions is designed to support the transfer of error self-regulation skills for the EBL intervention. This activity involves the person learning to complete of a set of errands within the home or community that are selected with consideration of participants’ goals and interests (for example, to increase independence in the home or community). The contrasting techniques of the EBL and EBL training procedures are shown in Table 2.

**Table 2:** Overview of error-based and errorless learning training techniques

**Session**	**Error-based learning (EBL)**	**Errorless learning (ELL)**
Session 1: role reversal or modeling	Role reversal: therapist makes a number of errors across all steps of the activity (for example, incorrect sequence, omitting a step).	Modeling: therapist describes out loud and models correct performance of each action during all activity steps.
	Participant checks the instructions to identify the therapist’s errors, pauses the task and describes the corrective action (with prompts if needed).	Participant reads instructions and observes therapist’s correct actions on each step.
	Post-task review of errors during each step and corrective strategies.	Post-task review of the correct performance on each step.
	Participant completes the activity according to the EBL procedures with post-task discussion of errors and their significance (for example, memory problems).	Participant completes the activity steps according to the ELL procedures with post-task discussion reinforcing error-free performance.
Sessions 2 to 3, 5 to 7: skill practice	Participant previews the task and makes self-predictions of possible errors for each step and planned strategy use (for example, use timer for cooking).	Therapist reviews the activity with the participant and breaks each step into smaller sets of action.
	Participant follows activity instructions with the therapist observing, but not directing his/her actions.	Therapist initially models each action (for example, measure the rice) and participants copy the action.
	When an error is observed the therapist delays responding for up to 10 seconds to allow participants to self-correct the error.	Therapist anticipates errors and provides a high level of cuing to guide participant’s actions to avoid opportunities for making errors.
	If an error is not self-corrected, therapists provide a non-specific prompt (‘Can you stop and check what you need to do’).	If an error occurs, the correct action is modeled and practiced until performance is error free.
	Post-task self-evaluation of performance with goals set to improve in target areas.	Post-task positive reinforcement for correct performance.
Sessions 4 and 8: skill mastery	Pre-task discussion and on-task prompting targets participant’s awareness and correction of any errors on the activity.	Pre-task review and repeated practice and reinforcement of correct actions within each step.
	Therapist systematically fades prompts to support independent and self-directed checking and strategy use.	Therapist maintains a high level of cuing to ensure error-free performance and habits.

### Intervention delivery

To minimize the potential effects of clinical bias and inadvertent contamination between the two interventions on the study outcomes [28], all therapists (two at each site) will administer each intervention in a randomized crossover fashion. Specifically, for the first half of the intervention study, one therapist will administer EBL and one therapist will administer ELL. For the last half of the intervention study, the therapists will switch over to administer the other type of intervention. Hence, therapists will administer both types of intervention, with a switch over approximately half way through the study. They will receive specialized training in the intervention type they are just about to administer. Using this approach, therapist effects will be balanced across the interventions [28]. Treatment fidelity will be monitored using a checklist based on Borelli’s framework [29,30]. Sessions will be audiotaped to enable therapists’ adherence to the treatment protocol to be examined for a random sample (20%) of sessions by experts who are independent of the study [30].

### Measures

The primary and secondary outcome measures and the timings of assessment are outlined in Table 3. The primary outcome measure assesses generalization of error self-regulation skills to a task distinct from but related to training (that is, meal preparation). Secondary outcome measures assess self-monitoring and self-regulation skills on a task unrelated to training, awareness of deficits, behavioral competency, role participation and supportive care needs.

**Table 3:** Summary of outcome measures and the timing of assessment

**Outcomes measures**		**Pre-intervention use**	**Post**-**intervention use**	**6-month follow-up**
Primary	Cooking task	X	X	
Secondary	Zoo map test	X	X	
	Patient competency rating scale	X	X	X
	Awareness questionnaire	X	X	X
	Sydney psychosocial reintegration scale	X	X	X
	Care and needs scale	X	X	X

#### Primary outcome

The Cooking Task [21] is a 1-h naturalistic assessment task that involves independently baking a cake and making an omelet for two people. The task involves standard instructions, recipes, equipment and scoring. The total error score encompasses five error types and is the main score for analysis. Inter-rater reliability (>0.80 for all error types), test-retest reliability (*r* = 0.89), discriminant and convergent validity have been reported [21,31]. Audiovisual recording will be rated by blind assessors with inter-rater reliability examined on 20 % of randomly selected DVDs.

#### Secondary outcomes

The Zoo Map Test from the Behavioural Assessment of Dysexecutive Syndrome [32] is a standardized neuropsychological test which examines the ability to plan, monitor performance and follow rules. In part 1, participants are asked to independently formulate and implement a plan to follow a route through a map without breaking specific rules. In part 2, participants follow a predetermined plan to complete the route. Although the profile score (1 to 4) will be calculated, the part 1 score (sequence score minus total errors) is the main index for analysis because this more directly assesses self-monitoring and self-regulation skills. The Zoo Map Test has been found to be a reliable and ecologically valid measure of planning and self-regulation [32,33].

The PCRS [25] is a well-established measure of awareness deficits and behavioral competency after TBI. The 30 items on the PCRS relate to participants’ current level of competency on activities of daily living (for example, meal preparation), interpersonal skills (for example, initiating conversations), behavioral (for example, controlling temper) and emotional function (managing depression) and cognitive abilities (for example, remembering conversations). Items are rated on a five-point Likert scale (1 = ‘can’t do’, to 5 = ‘can do with ease’). The discrepancy score between self-ratings and informant ratings on the PCRS is calculated to provide an index of self-awareness. Relatives’ ratings on the PCRS will assess skills generalization or broader behavioral competency. The PCRS has good test-retest reliability (*r* >0.85) and internal consistency (α >0.90) and established validity, including sensitivity to change [34].

The Awareness Questionnaire (AQ) [35] is a 17-item scale that assesses awareness of deficits across sensory, physical, cognitive, and behavioral domains. Unlike the PCRS, ratings on the AQ compare participants’ post-injury abilities on each item to their pre-injury functioning (that is, 1 = much worse, 3 = about the same, 5 = much better). Positive discrepancy scores (participant minus relative ratings) indicate impaired self-awareness. The AQ has sound psychometric properties [35] and has demonstrated sensitivity to change in the context of awareness interventions [18].

The Sydney Psychosocial Reintegration Scale (SPRS; [36]) is a 12 item questionnaire that assesses level of psychosocial reintegration, or the ability of a person with brain injury to resume valued societal roles, including those of worker, driver, parent and spouse. The scale comprises three domains, and four questions for each domain. The three domains of the SPRS are: occupational activities, interpersonal relationships, and independent living skills. Excellent psychometric properties have been reported for the SPRS, including internal consistency (α = 0.90), inter-rater (intraclass coefficient (ICC) = 0.95) and test-retest (r = 0.90) reliability, construct validity, sensitivity, and support from Rasch analysis [36,37].

The Care and Needs Scale (CANS; [38]) measures the extent to which an individual can be left alone in the community, using a rating scale of 0 (‘Can live in the community, totally independently, does not need contact’) to 7 (‘Cannot be left alone, needs nursing care, assistance, and/or surveillance 24 hours per day’). The weekly hours of assistance required are also recorded. Psychometric properties, including inter-rater (ICC = 0.93 to 0.96) and test-retest (ICC = 0.98) reliability and construct, concurrent and predictive validity have been reported [38,39].

Ratings on the SPRS and CANS will be made by the blind assessors based on an interview with the relative (SPRS), and the participant and the relative (CANS) in accordance with the authors’ guidelines [36-39].

### Sample size

Our previous RCT findings [14,15] indicated that individualized EBL training was more effective than another active intervention in improving awareness of deficits and behavioral competency between pre-intervention and post-intervention assessment (group × time interaction effects: partial η^2^ = 0.061 to 0.13). Based on a similar design and approach to statistical analysis, a conservative power analysis was conducted using an estimated medium effect size (partial η^2^ = 0.061, PCRS discrepancy score), alpha level of 0.05, and power of 0.8. According to G*Power [40], a sample size of *n* = 123 is required to detect significant effects. Due to possible participant dropout (estimated as 10% from the previous RCT [14]), a total sample size of *n* = 135 will be required.

### Statistical analysis

Data will be screened to ascertain any state/site or therapist effects or the effects of demographic and clinical factors on outcome, with any significant variables treated as covariates in respective analyses. Participant dropout will be managed on an intention-to-treat basis using the last observation carried forward method, and participants will be analyzed in the group to which they were allocated, irrespective of compliance or withdrawal from treatment [41]. To compare the efficacy of EBL and ELL for improving error self-regulation skills, analysis of covariance (ANCOVA) will be conducted using endpoint means and adjusting for baseline scores for the primary outcome (Cooking Task errors) and secondary outcome (Zoo Map part 1). The estimated effect size and its precision will be examined using a 95 % CI for the mean difference. Mixed 2 × 3 analysis of variance (ANOVA) procedures (grouped by time: pre-intervention, post-intervention and follow-up periods) will examine the impact of each intervention on awareness of deficits, behavioral competency, role functioning and support needs. In the latter analyses, interaction effects will be examined to determine whether EBL is associated with greater gains than ELL relative to pre-intervention functioning. Hierarchical regression will be used to determine whether pre/post changes in awareness, error self-regulation and behavioral competency predict long-term outcomes on the SPRS and CANS after controlling for pre-intervention functioning.

## Discussion

TBI is a leading cause of lifelong disability that mainly affects young people at the start of their independent and working lives. Impairments in self-awareness and self-regulation greatly undermine people’s ability to live independently and re-enter the workforce. By comparing the efficacy of EBL and ELL this project will determine the benefits of training internal self-regulation strategies after severe TBI. The findings are expected to have important theoretical and practical implications for treatment approaches that can potentially be used to enhance individuals’ everyday functioning and reduce the personal and social burden of TBI in the community.

Traditionally, rehabilitation trials have focused on increasing or decreasing target behaviors on a specific task (for example, remembering steps to pay a bill). An issue that has typically been neglected is whether training generalizes beyond the therapy context [11]. Lack of skills generalization poses one of the biggest barriers to successful outcomes of rehabilitation after a TBI. This study seeks to determine whether it is beneficial for people with severe TBI to make errors as they learn or relearn skills in rehabilitation. It will also examine whether immediate post-training gains translate to improved long-term functional outcomes, which is the ultimate goal of rehabilitation. In doing so, the project will clarify the role of errors in task learning and generalization. Addressing these research priorities represents the key innovations of the present RCT, and the findings are expected to revise and guide clinical practice in neurorehabilitation.

The main methodological challenge in this study relates to retention of participants over the 6-month follow-up period. To enhance retention success we will obtain multiple points of contact from participants. The use of telephone-based assessment has been found to be well accepted by people with TBI and their relatives, and is more effective and economical than postal return of questionnaires and home-based assessments respectively [42]. Overall, the feasibility of the assessment and intervention procedures employed in this study is supported by our pilot research [15,16,18].

## Trial status

Recruitment for the study commenced July 2013.

## Abbreviations

EBL: Error-based learning; ELL: Errorless learning; TBI: Traumatic brain injury.

## Competing interests

The authors declare that they have no competing interests.

## Authors’ contributions

All authors contributed intellectually to the study design and were involved in the drafting of the manuscript and/or critically revising the intellectual content. TO had the lead role in preparing the manuscript, and conceived the study design, methodology and intervention procedures. JF contributed to the conceptualization of the study, research design and development of intervention procedures. RT participated in the study design and selection of outcome measures. DHKS contributed to the methodology and statistical analysis. JG participated in participant recruitment and screening procedures for the Brisbane-based participants. JS contributed to the ethics application and development of participant recruitment and screening procedures for Sydney-based participants. AL-B contributed to staff recruitment, training and supervision of staff and participant recruitment in Sydney. MK participated in the research design and assisted with participant screening and recruitment in Brisbane. MC provided input on the administration and scoring of the primary outcome measure and intervention delivery. All authors read and approved the final manuscript.
